# Placental pathology reports: A qualitative study in a US university hospital setting on perceived clinical utility and areas for improvement

**DOI:** 10.1371/journal.pone.0286294

**Published:** 2023-06-08

**Authors:** Kelly Gallagher, Jane-Frances C. Aruma, Christiana N. Oji-Mmuo, Jaimey M. Pauli, William M. Curtin, Jeffery A. Goldstein, Heather L. Stuckey, Alison D. Gernand

**Affiliations:** 1 Ross and Carol Nese College of Nursing, The Pennsylvania State University, University Park, Pennsylvania, United States of America; 2 College of Medicine, Pennsylvania State University College of Medicine University Park Campus, Hershey, Pennsylvania, United States of America; 3 Division of Neonatal-Perinatal Medicine, Department of Pediatrics, Penn State University College of Medicine, Hershey, Pennsylvania, United States of America; 4 Division of Maternal-Fetal Medicine, Department of Obstetrics and Gynecology, Penn State University College of Medicine, Hershey, Pennsylvania, United States of America; 5 Division of Maternal-Fetal Medicine, Department of Pathology and Laboratory Medicine, Penn State University College of Medicine, Hershey, Pennsylvania, United States of America; 6 Department of Pathology, Northwestern University Feinberg School of Medicine, Chicago, Illinois, United States of America; 7 Department of Medicine, Penn State University College of Medicine, Hershey, Pennsylvania, United States of America; 8 Department of Nutritional Sciences, The Pennsylvania State University, University Park, Pennsylvania, United States of America; University of Illinois Medical Center at Chicago: University of Illinois Hospital, UNITED STATES

## Abstract

**Objective:**

To explore how placental pathology is currently used by clinicians and what placental information would be most useful in the immediate hours after delivery.

**Study design:**

We used a qualitative study design to conduct in-depth, semi-structured interviews with obstetric and neonatal clinicians who provide delivery or postpartum care at an academic medical center in the US (n = 19). Interviews were transcribed and analyzed using descriptive content analysis.

**Results:**

Clinicians valued placental pathology information yet cited multiple barriers that prevent the consistent use of pathology. Four main themes were identified. First, the placenta is sent to pathology for consistent reasons, however, the pathology report is accessed by clinicians inconsistently due to key barriers: difficult to find in the electronic medical record, understand, and get quickly. Second, clinicians value placental pathology for explanatory capability as well as for contributions to current and future care, particularly when there is fetal growth restriction, stillbirth, or antibiotic use. Third, a rapid placental exam (specifically including placental weight, infection, infarction, and overall assessment) would be helpful in providing clinical care. Fourth, placental pathology reports that connect clinically relevant findings (similar to radiology) and that are written with plain, standardized language and that non-pathologists can more readily understand are preferred.

**Conclusion:**

Placental pathology is important to clinicians that care for mothers and newborns (particularly those that are critically ill) after birth, yet many problems stand in the way of its usefulness. Hospital administrators, perinatal pathologists, and clinicians should work together to improve access to and contents of reports. Support for new methods to provide quick placenta information is warranted.

## Introduction

In the clinical setting, placental pathology evaluation is currently used to explain adverse pregnancy outcomes, to provide information to guide counseling and improve management for future pregnancies [1] and may inform care in some neonatal settings [2]. How clinicians use placental pathology in their daily workflow and patient care is largely undescribed. Placental pathology is a valuable tool in understanding pregnancy events. The usefulness of pathology examination in stillbirth, for example, is widely documented [3–6] and when completed, is estimated to decrease unexplained stillbirth by 83% [1]. Placental pathology can also provide helpful information to counsel families on risks for subsequent pregnancies [7, 8]. Over the last decade, there has been a steady stream of research that links placental conditions with newborn health [9–13] and long-term maternal health [14–17] which could also inform future health risks and be used in patient counseling. Additionally, multiple epidemiological studies link placental characteristics with long-term future disease states in offspring [18–25].

College of American Pathologists’ (CAP) guidelines provide lists of maternal, fetal and placental conditions which should prompt clinicians to consider a full pathology exam of the placenta [26]. It is difficult to know how consistently these guidelines are used throughout the United States, but two studies suggest a wide range of pathology utilization, with one study estimating that out of placentas that should have been sent to pathology, only 49% were actually sent [27] and another study estimating that 82% of placentas were sent appropriately [28].

Guidelines for placental pathology examination currently rely on specific pregnancy, fetal or placental conditions to prompt an examination. This is largely due to the idea that there will be a low yield of findings in normal, uncomplicated pregnancies and that the cost of such an exam would be unrealistic [1]. The result of this approach is that at least some placentas with significant clinical findings are overlooked [29].

In an ideal situation, all placentas could be evaluated for potential clinically significant findings. Recent advances in artificial intelligence and deep learning make the possibility of rapid placental examination for triage of placentas possible at every birth and our research group has been working towards this goal [30, 31]. As these technological advances increase the potential speed of results, it becomes important to understand current practice patterns for placental pathology and what rapid information from the placenta is desired. To this end, our aims were to 1) assess clinician use of placental pathological diagnoses in clinical care of postpartum mothers and newborns, and their needs for better understanding of these diagnoses and application to clinical care, and 2) explore with maternal and neonatal care clinicians what information could improve clinical care for mothers and newborns that might be ascertained from placental data.

## Methods

This qualitative study employed in-depth, semi-structured interviews with maternal, neonatal, and pediatric clinicians at Penn State Milton S. Hershey Medical Center, an academic medical center with a level IV neonatal intensive care unit (NICU) located in central Pennsylvania in the United States. Email invitations were sent by study team members and administrative support staff to every member of the maternal, neonatal, and pediatric clinical staff (n = 47), that is, everyone who attends deliveries or cares for mothers or newborns postpartum. The first request yielded approximately half of the interested participants, and two subsequent email invitations yielded the remainder. Twenty clinicians responded affirmatively and 19 provided verbal consent and were interviewed; one could not find a suitable time to be interviewed. Inclusion criteria were: (1) licensed physicians not in training or advanced nurse clinicians including certified nurse midwives (CNM) and women’s health nurse practitioners; (2) currently providing clinical care for mothers or newborns at or after birth; (3) responsible for ordering placentas for pathology and/or interpreting pathology reports; (4) employed at Penn State Health Milton S. Hershey Medical Center; and (5) willing to be recorded.

### Procedures

**Fig 1:**
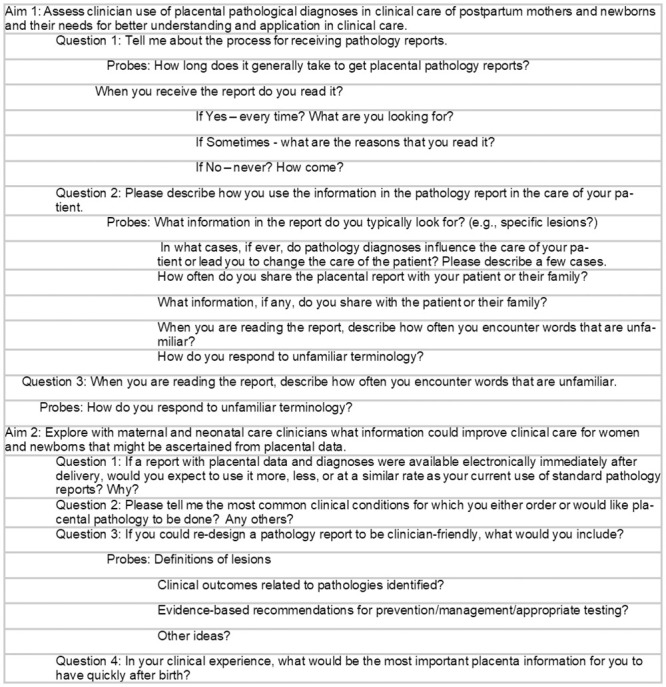
Interview guide.

Individual interviews were scheduled based on clinician availability and conducted over the Zoom conference platform from November 2019 to May 2020. A semi-structured interview guide (Fig 1) which was pilot tested before the study was used to collect information related to the study aims. We interviewed maternal and neonatal clinicians as well as family medicine staff who care for both. The interview questions were the same for both groups, but the background and demographic information questions were slightly different to capture information about those clinicians’ specific experiences. Family medicine participants were asked to reflect on their experiences caring for both mothers and babies. The interviews consisted of four background/demographic questions, and seven questions to explore the two aims. At the beginning of every interview, the consent document was read to participants, and verbal consent was obtained and recorded before the interview began. All participants were informed that their participation was voluntary and that they could decline any questions or stop the interview at any time, and all agreed to participate in audio-video recording. The interviews lasted from 20–30 minutes. No repeat interviews were needed.

All interviews were conducted and transcribed by the lead author (KG), a PhD prepared nurse-midwife working in an academic, non-clinical faculty position. Participants were informed of the lead author’s educational background and current employment status in the introductory email and her clinical background was discussed with all participants at the beginning of the interview. Video and transcript data were stored on a secure, password protected cloud service. No additional notes were taken; all data were collected by interview. Clarifications from participants, when needed, were captured during the interview and transcripts were not returned to participants. This study received Institutional Review Board approval from The Pennsylvania State University (STUDY00015903).

### Data analysis

**Fig 2:**
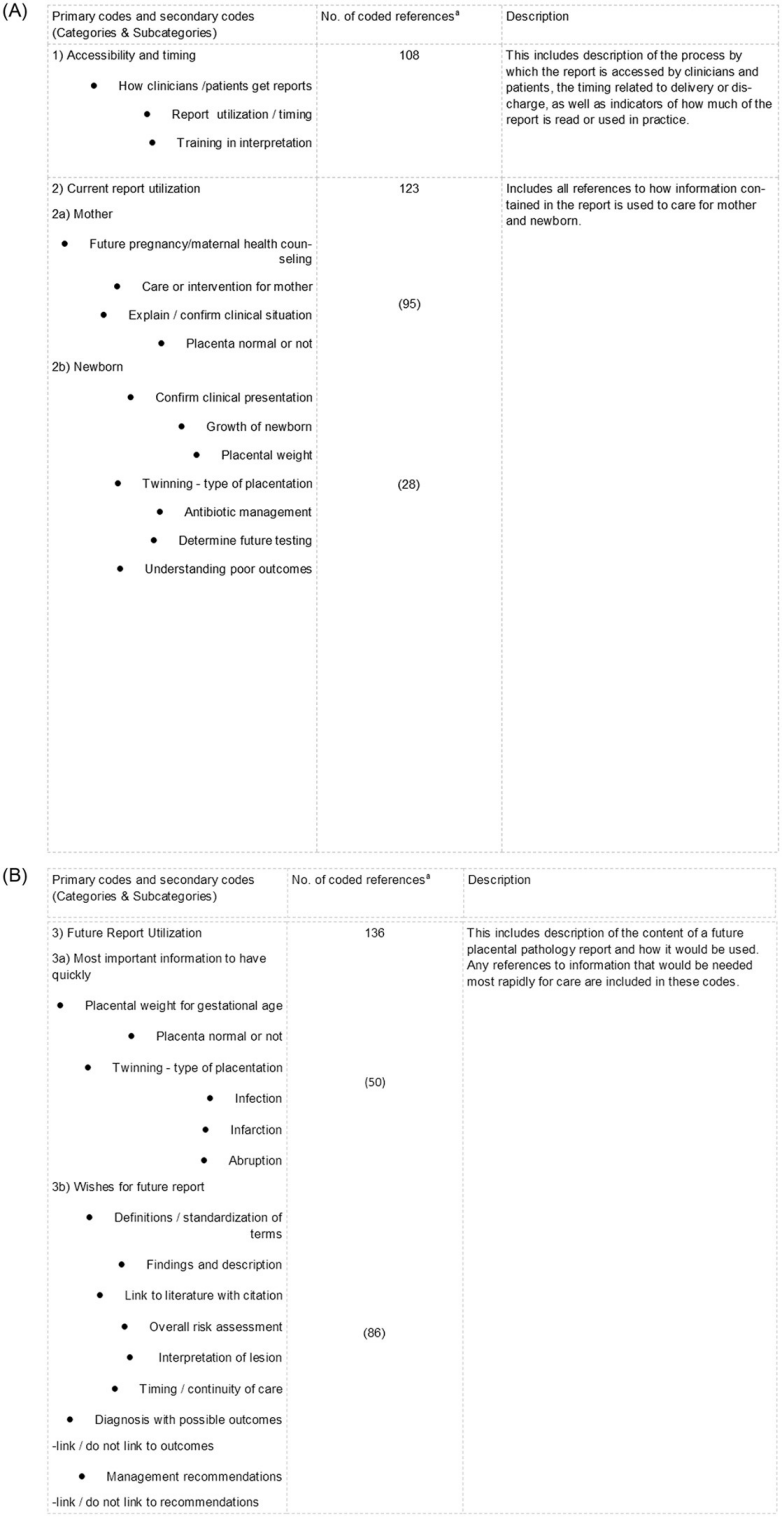
A and B. Example of code definitions. ^a^ Number of times the code was identified in the transcripts as evidenced by specific discussion of secondary codes.

Transcripts were analyzed after all interviews were completed using descriptive content analysis [32]. Interviews were read by three authors (KG, JA, AG) and codes were identified using a data-driven approach [33]. A codebook was created, and each code was defined (Fig 2A and 2B) to enhance consistency among the coders. KG and JA conducted all coding using NVivo Version 12 (QSR International Pty Ltd.). The two coders independently coded 20% of the transcripts, and after satisfactory agreement was reached (k = 0.80), one coder (JA) completed the coding for the remaining transcripts. Participant feedback on findings was not used for this study. Practice type was used to aid interpretation: clinicians were grouped by those that cared for the mother, infant or both, or by specific training (OB/GYN and Family Medicine, MFM, CNM/NP, Pediatricians, Neonatologists). In some instances, the type of clinician is identified but, for most, “clinician” was used to label the data to protect the identity of the participants. Our intention was to interview every clinician who expressed interest in the study, regardless of the possibility of data saturation, and redundancy of ideas and comments by the participants was observed during coding.

## Results

Nineteen clinicians were interviewed: seven maternal, nine neonatal or pediatric, and three for both maternal and neonatal/pediatric (family medicine). The participants represented 40% of the combined maternal, neonatal and pediatric clinicians who work in the newborn nursery and 47% of the maternal and NICU clinicians at Penn State Hershey (Table 1). The clinicians were generally experienced, with a mean of 15 years (range 1–34 years) in practice across specialties (Table 2). Participants had a wide range of number of encounters with placental pathology reports.

**Table 1:** Clinicians interviewed by type and percentage of workforce.

Type of clinician	Number invited	Number interviewed	% of hospital workforce
OB/GYN and Family Medicine	12	4^a^	33%
Maternal Fetal Medicine	6	3	50%
CNM/NP	5	3	60%
Pediatrician	11	2	18%
Neonatologist	13	7	54%

Abbreviations: OB/GYN, obstetrician/gynecologist; MFM, maternal fetal medicine specialist; CNM, certified nurse-midwife; NP, nurse-practitioner

^a^OB/GYN (n = 1) and family medicine (n = 3) were combined because they are MD providers caring for similar patient risk levels in this setting.

**Table 2:** Number of years in practice and number of placental reports encountered per year.

Clinician characteristics	OB/GYN and Family Medicine (n = 4)	MFM (n = 3)	CNM/NP (n = 3)	Pediatrician (n = 2)	Neonatologist (n = 7)
	Mean (range)				
Years in practice	14.3 (5–34)	16 (7–21)	19.7 (15–23)	15.5 (10–21)	10.9 (1–30)
Estimated number of births attended per year	45 (20–75)	50	87^a^ (50–125)	N/A^b^	N/A^b^
Estimated % of encounters where placental pathology is ordered or available	47% (25%-80%)	48% (20%-75%)	65% (30%-95%)	6% (1%-10%)	20% (0–75%)
Number of placental pathology reports encountered in a year	31 (10–60)	103 (10–200)	78 (50–100)	3 (0–5)	31 (0–87)

Abbreviations: OB/GYN, obstetrician/gynecologist; MFM, maternal fetal medicine specialist; CNM, certified nurse-midwife; NP, nurse-practitioner

^a^Represents CNM attended births, NP’s do not attend births.

^b^Pediatric clinicians may attend births for resuscitation purposes, but number per year not assessed.

Participants were asked if it were ever the case that they would like to see a pathology report, but it wasn’t available to them. Ten participants answered “yes”–OB-GYN (n = 1), MFMs (n = 2), CNM/NP (n = 1), Neonatologists (n = 6), and nine answered “no”–Family Medicine (n = 3), MFM (n = 1), CNM/NP (n = 2), Pediatricians (n = 2), Neonatologist (n = 1). Participants were also asked if they were aware of a standard operating procedure specifying when to send a placenta to pathology for examination. Eight participants were aware of a standard operating procedure for sending a placenta to pathology and eleven were either unaware or believed that there was not a standard operating procedure available. Participants also recounted varying estimates from 48 hours to 2 weeks for how long it took to receive a pathology report. Unfamiliar terminology in reports was a frequent occurrence: eight participants said it happened frequently and five said at least sometimes, while only four participants related never encountering unfamiliar terminology. Two participants reported that they did not read enough placental pathology reports to be able to answer the question.

### Overview of themes

Through qualitative thematic analysis, four main themes (and three subthemes) were identified to answer the study aims. Theme 1: the placenta was sent to pathology for consistent reasons; however, the pathology report was accessed by clinicians inconsistently. Subthemes were that the report a) was difficult to find; b) was difficult to understand, and c) didn’t come fast enough. Theme 2: clinicians valued placental pathology for explanatory capability as well as for contributions to current and future care. Theme 3: rapid placental exam would be helpful in providing clinical care. Theme 4: placental pathology reports that connect clinically relevant findings and that are written with plain, standardized language are preferred.

#### Theme 1: The placenta is sent to pathology for consistent reasons; however, the pathology report is accessed by clinicians inconsistently

Many clinicians reported that there was a standardized list available to them on the labor and delivery unit that delineated common conditions for which the placenta should be sent. Others weren’t sure if there was a specific list but reported that there was a common understanding on the unit for when placentas should be evaluated, such as for preterm birth or in the circumstance of maternal preeclampsia. Accessing the placental report consistently proved to be more challenging. In the timeframe between delivery and the placental pathology report posting to the chart, clinicians reported it was not always immediately clear if the placenta had been sent to pathology, nor where this information was located in the chart. Some clinicians reported that they knew it was sent by looking in the orders; others would read the delivery note to see if it was mentioned. Three subthemes related to access to placental report information are detailed below:

*Subtheme 1a*: *Difficult to find*. Clinicians reported that to find a placental pathology report, one must look for it. Neonatal clinicians certainly must seek out the report if it is desired. For maternal clinicians, the person who ordered the pathology report at delivery may or may not receive the final report in their electronic chart inbox because the placenta report will be sent to the patient’s admitting physician, and these are not always the same person. This is unlike other labs which are sent back to the ordering clinician. Then the result is archived in the mother’s electronic medical record and available for those who seek it.

*It’s a mess*. *So*, *the placenta gets ordered by whoever of the residents did the delivery essentially*. *Because the way our system works*, *that resident doesn’t actually get the report back*. *It gets assigned to whatever attending was the admitting physician*. *It’s very*, *very strange*. *So unless you go look for it*, *you’re probably not going to get it*. *If you see the patient postpartum*, *we usually look for it postpartum*, *but almost always you’re not going to be the one that ordered it*, *so it’s not going to pop up into your box to review*.

–Obstetrician

Clinicians also reported that they didn’t always know automatically if a report was requested. There was no easy place to look in the chart and know that it was ordered, sent, and pending, so there were times when the clinician thought they would get a placental pathology report, when in fact it was never ordered. More than one clinician noted that the placenta is fetal in origin, and that they felt it should be in the baby’s chart, or perhaps even located in both the maternal and neonatal chart.

*If it’s able to come to the infant’s chart that will enhance a pediatrician’s access and viewing of it*, *but I know sometimes that it is not always allowed because it’s mom’s material*. *It’s mom’s specimen*, *and so it may need to stay in maternal chart*.

–Pediatrician

*Subtheme 1b*: *Difficult to understand*. Clinicians did not always understand the information in the report, nor feel they were adequately trained to interpret the report. A few clinicians reported improvement of understanding with time and exposure to reports.

*I mean*, *honestly*, *I didn’t get a lot of training in residency on how to interpret a placental report*, *and it’s not something that has come up a lot since I started practicing*.

–Clinician

*I’m going to have to say probably most*, *the majority of the time there’s something in there that I don’t fully understand*, *Okay*, *being that I’m*, *not a pathologist*.

–Clinician

*Subtheme 1c*: *Doesn’t come fast enough*. Some clinicians reported engaging regularly with placental pathology reports, especially the maternal fetal medicine and the neonatology experts. Other clinicians, however, noted that the placental pathology result is rarely returned in enough time to impact their workflow. Clinicians summarized the issue succinctly, saying, “*honestly*, *I don’t do much with the information since it’s after the fact*” and “*by the time I have the report the mom and baby have been discharged*.”

*Because it is not available in the time I would need to use the information to take care of my patients*, *it just never comes to my mind to consider it*…*it’s not information that is readily available to us in the time that we would need*.

–Pediatrician

#### Theme 2: Clinicians value placental pathology for explanatory capability as well as for contributions to current and future care

Many clinicians detailed several types of encounters where it was helpful to have placental pathology information. These ranged from instances of growth restriction to maternal preeclampsia, and in rare cases of fetal death. In these situations, clinicians reported using placental pathology to help them understand pregnancy and delivery events, and to allow them to reconcile their thoughts about what occurred in the pregnancy or delivery with placental information. One common scenario mentioned was in the care of the baby who is smaller than expected for its gestational age.

*The information can also be useful if we’re looking for causes for growth restriction*. *If the placenta can be a cause of it*, *we feel more comfortable in assigning that etiology to the growth restriction and don’t necessarily have to continue to worry about or track down metabolic or genetic disorders*.

–Clinician

Other clinicians reported looking at the pathology to understand the clinical reason for a demise or poor neonatal outcome. They noted that results from placental pathology were also helpful for planning care for a future pregnancy and influenced their counseling for patients about the importance of preconceptual care.

*I think it* [placental pathology] *influences it* [*care*] *in the sense that if I’m doing preconception counseling on a woman who had an IUFD* [*intrauterine fetal death*] *or something like that*, *I will use all the information including placental pathology*, *to help her with the next pregnancy*. *I don’t think it changes much*, *but it adds to the counseling*.

–Clinician

*And then long term*, *some very specific placental lesions may have a risk for recurrence and so I look for those as well*, *in a future pregnancy*.

–Clinician

When available, placental pathology can be useful for antibiotic management. Many NICU clinicians noted that, while not always available quickly enough, there were many times when a placental pathology report directly impacted care for a baby under their care.

*We frequently start empiric antibiotics in babies because of their clinical presentation*. *The placenta may in some instances factor into a decision about how long we continue antibiotic therapy*.

–Clinician

*When there is funisitis*, *meaning an infection on the baby’s side that we see on the placenta*, *and if the baby had any sort of clinical suspicion*, *then I would lean more towards treating the baby with antibiotics longer*. *So sometimes the description of funisitis pushes me to continue antibiotics longer than I would have otherwise*.

–Clinician

#### Theme 3: Rapid placental evaluation would be helpful in providing clinical care

Clinicians reported that if they could have access to at least some placental data quickly, perhaps in the first hours after birth, this could impact clinical care immediately. Many clinicians paid attention to placental weight, which helps them quickly consider the newborn’s size.

*I think the weight of the placenta matters to me*. *Usually I think of placenta as being a third of the baby’s weight*, *so sometimes I use that to gauge whether the placenta was big or small*.

Clinician

Additionally, infection and infarction were frequently mentioned as information that would be impactful in the first hours of birth, with clinicians saying they would like to see, “*a weight*, *and if there is an infection*, *and infarction*” and “*infection and infarction/abruptions*.*”*

Completeness of the placenta was also mentioned as a helpful piece of information to have rapidly by maternal care clinicians. While delivery attendants visually inspect the placenta after birth, in some cases it is difficult to determine if the placenta is complete without expert assessment. Clinicians said that clear information on incomplete placenta would factor into their postpartum maternal surveillance.

*The appearance tells me whether or not we need to keep an eye on the patient for postpartum hemorrhage*. *If I suspect that there’s a little bit of a placental piece that’s missing*, *then I know that we need to watch for hemorrhage*, *or if it looks like there’s a piece that may have been missing*, *we can watch for infection*.

–Maternal care clinician

Infection information from the placenta is already used by NICU clinicians when available, and would be valuable to know immediately for maternal care, too, when possible. Clinicians imagined nuances of starting or stopping antibiotics, and even using rapid placental pathology information in conjunction with blood culture results.

*If there is a way that information about infection can help us stratify the newborn’s risk*, *that would be most helpful*, *because that’s what we’re usually trying to figure out in the first 24 hours*. *Does the baby need antibiotics*? *Can we just monitor them*?

–Clinician

*It would help me make a decision about continuing antibiotics*, *particularly if the blood culture is negative and the baby looks or acts like a septic baby*. *Then I would*, *if the pathology supports the infection*, *inflammation*, *then I would treat the infection as such*.

–Clinician

Finally, in imagining a future where some placental information would be available immediately, some clinicians reported that a quick assessment that informed an overall risk assessment would be useful.

*I would like a general idea if the placenta looks like it was essentially normal… and I wouldn’t need detail*, *but you know*, *to have something that says this doesn’t look quite right*.

–Clinician

#### Theme 4: Placental pathology reports that connect clinically relevant findings and that are written with plain, standardized language are preferred

Clinicians reported a need for information about placental pathology that is written in a uniform manner with standardized terms and ideally that connects findings in the placenta to clinically relevant issues. Clinicians lamented that different pathologists use different terminology, and they noted that standardized terms would be helpful.

*I’ve noticed that different pathologists will use different terminology for basically the same finding*, *and so standardization of the terms that they’re using would be fine*. *Part of me would like it to be more plain language*, *so a non-pathologist would be more readily able to read it*. *But at the same time*, *I kind of feel a little guilty about that*, *like I’m a doctor*, *I should probably know what these words mean*. *You know*, *we’re not writing a children’s book*, *but I think at least standardization would be a little bit helpful*, *so that I’m not encountering a new way to describe the same problem*.

–Clinician

Major placental findings and the potential clinical relevance were reported frequently as useful information to have rapidly.

*I might ask for some pathologic correlations*, *that if certain histologic findings*, *if those were associated with certain prenatal conditions or infant conditions*, *that’d be great*, *you know*, *sort of a little minimized pathology textbook*.

–Clinician

Some also recommended a report similar to the types found in radiology, where potential clinical considerations are highlighted.

*I always love when our specialists*, *radiologist or pathologist*, *include a line at the bottom that says*, *you know*, *these are the findings and we see on the slide or on the studies*, *and it could indicate this clinical scenario and then they detail consider this*, *this*, *and this as your clinical entity*. *I really find that helpful*, *especially for things that aren’t occurring very often or variants that I’ve never seen before*. *I really appreciate that point in a direction*.

–Clinician

Another clinician recalled pap smear reports and how these reports also help to direct clinicians to the right care.

*Pap smear reports have*…*they don’t tell you how to manage the patient*. *However*, *they do say negative*. *They do say high risk*. *They do* [contain] *certain terminologies that you would probably need*, *so I would really appreciate that*. *Because right now it* [information from placental pathology reports] *is truly very generic*.

- Clinician

While most clinicians stated that placental findings connected to clinical outcomes would be helpful, there were a few clinicians who had reservations with this approach. The concern for these clinicians was that if certain outcomes were suggested and then misinterpreted by clinicians, then more harm than good might be done.

*I don’t think that’s as helpful*, *because I think that the pathology report is a data piece and it might*, *ah*, *I mean*, *I*, *I think it should be used by the appropriate providers*, *but I don’t*, *if you suggest potential outcomes*, *I think you could potentially go down other pathways that are maybe not as beneficial*.

- Clinician

*I’m always leery about making things real cookie cutter because I feel like it gives a false sense of security*. *I think using the syncytial knots as an explanation*, *like explaining what that means and then saying like this is oftentimes seen in cases of hypertensive disorders of pregnancy and then leaving it at that*. *Yeah*, *because I’m kind of concerned that if you start saying like*. *Ah*, *your patient may have blah blah blah blah blah like that’s*, *it goes down a really slippery slope*.

- Clinician

## Discussion

Among US clinicians in a mid-sized university hospital setting, this qualitative study found that clinicians value placental pathology; that they would like to have access to the information sooner; and that they would prefer the information to be provided in standardized language and linked with clinical information that might be relevant. For some clinicians, placental information influenced the care they gave to their patients, for example in NICU situations and in providing counseling for future pregnancies. Other clinicians used placental pathology information to explain the clinical situation that they were seeing. This is consistent with what has been reported in the literature about the clinical utility of placental pathology [1, 2].

Maternal clinicians used the information from the placental pathology report to explain clinical events and counsel for future pregnancies, if they used the information at all. Neonatal clinicians also used placental pathology information to help explain the clinical situation they were encountering but were also much more likely to use the information for clinical care, specifically antibiotic management. This research reaffirmed that placental pathology information is frequently not available fast enough to even be considered as part of information relevant to the immediate care of the patient, except in the case of the NICU patient who is often under care for extended periods of time.

Previous research suggested that the language used in placental reports is difficult to understand, and may impose barriers to care [34]. This qualitative study also found that clinicians frequently find the terminology in placental pathology reports confusing. Some progress is already underway or achieved to realize standardized terminology with the creation and publication of the Amsterdam Placental Workshop Group Consensus Statement [35]. Additionally other research also recognizes the need for clinical translation of placental pathology findings and novel approaches to communicate placental findings to all stakeholders, including the patient [36].

Several clinicians reported challenges accessing placental pathology reports or knowing when reports were ready. For example, some clinicians thought the report should be in both the maternal and neonatal chart and may not know that in this hospital, the obstetric and neonatal charts are linked, so it is possible to move from one chart to the other. Specific reporting issues are likely to vary between electronic health record implementations. However, obstetric practice is characterized by 1) the unpredictability of labor onset, such that the prenatal clinician, the admitting clinician, and the delivering clinician frequently differ and 2) placenta reports are relevant to both the mother and the newborn(s). This differs from the usual laboratory practice where one specimen is linked to just one patient, which creates friction. Changes in test reporting and alerts may be needed to put reports in front of relevant providers in a timely fashion. Reporting systems for typical anatomic pathology specimens where the same surgeon will see a patient pre-operatively in the office, admit them to the hospital, and perform the surgery are a poor fit.

All participants reported they could imagine using placental information and incorporating it into their clinical care if they could get the information rapidly. Many clinicians reported that they could envision using rapid placental data for antibiotic management, both initiating and withholding and monitoring closely in the case of an infant with soft signs of infection but a negative rapid placental assessment. The placenta holds a wealth of information about the health of the pregnancy, intrauterine events, and future health of both mother and newborn, but it is underutilized.

Previous studies show that placental exams are not performed consistently even in the clinical situations for which it is indicated [27, 28], which may deny the potential benefits of placenta-informed care to patients. Even for placentas not included under the CAP guidelines, there is untapped potential when it comes to evaluation. Romero et al. studied placentas from pregnancies with normal outcomes and found 78% had lesions consistent with inflammatory or vascular lesions, many of which may be consistent with a sub-clinical infectious process not affecting the pregnancy or with labor [29]. However, in this same study of 944 placental samples from term gestations without complications, lesions concerning for maternal (7.4%) and fetal (0.7%) malperfusion were also present. What is needed is the ability to rapidly and at low cost evaluate all placentas to provide some information that is immediately useful for clinical care, identify placentas that would benefit from a thorough analysis, and that can provide information that ultimately can contribute to the growing body of research connecting placental features to clinical outcomes. Additionally, a standardized approach to placental assessment reporting that includes normal reference values and potentially links to relevant literature could improve clinician knowledge base and interpretation.

### Strengths and limitations

A strength of this study was including both maternal and neonatal clinicians who interact with placental reports, whereas other studies have focused on one or the other. We had good representation from both the maternal and the neonatal/pediatric work force. Overall clinicians were experienced but we were also able to capture a range of perspectives and experience levels, especially in the NICU. While clinicians reported sending placentas appropriately, there is no way to know if placentas were actually sent in all or most circumstances where it would be clinically indicated, and this is a limitation. Furthermore, this study took place in a single academic medical center, which quite likely differs from other sites in electronic medical record capabilities, practice patterns, and possibly in appreciation or knowledge base related to placental pathology.

## Conclusion

Our qualitative survey of 19 practicing maternal and neonatal clinicians identified several barriers to effective use of placental pathology in patient care: 1) technical challenges in finding and getting reports; 2) long turn-around-time between delivery and report production; 3) dense, inconsistent language in reports; and 4) poor documentation of links between placental pathologic findings and outcomes. Yet, clinicians wanted the pathology reports, particularly to manage antibiotics and plan future care needs, and said that rapid placenta reports at the time of birth could improve medical care. These findings support the need for improvements in medical software, such as making a clear link to placenta data from maternal or newborn charts, improvements in traditional pathology reports, and new tools to rapidly assess the placenta at birth. Future research should evaluate and quantify how such changes can improve health outcomes.

## Supporting information

S1 Data Data table of themes and sample quotes. (PDF)

S2 Data Coding structure summary. (PDF)
