# Supplementary material for: Placental pathology reports: A qualitative study in a US university hospital setting on perceived clinical utility and areas for improvement
Source: PLoS One. 2023 Jun 8;18(6):e0286294. doi: 10.1371/journal.pone.0286294 (PMC10249791; doi:10.1371/journal.pone.0286294)
Supplement: S1 Data — (PDF) [file pone.0286294.s001.pdf]

Aim 1: Assess clinician use of placental pathology diagnoses in clinical care of postpartum mothers and newborns and their needs for better understanding of these diagnoses and application to clinical care.	
**Theme 1:** The placenta is sent to pathology for consistent reasons, however, the pathology report is accessed by clinicians inconsistently due to a range of barriers.	
	**Supporting Quotes**
**Subtheme 1a:** Difficult to find	• the only way we would receive them would be if we had a particular concern and we went back into the maternal chart and searched out the path report in the EMR.<br>• it's a mess. So, the placenta gets ordered by whoever of the residents did the delivery essentially because the way our system works, that resident doesn't actually get the report back it goes to, somehow it gets assigned to whatever attending was the admitting physician. It's very, very strange. So unless you go look for it, you're probably not going to get it, so if you see the patient postpartum, we usually look for it postpartum, but almost always you're not going to be the one that ordered it, so it's not going to pop up into your box to review.<br>• sometimes the report can be excellent, but finding it in the medical record can be a challenge. You know that might be more the health system than the person you know who creates this report, but it needs to be easily retrievable and shareable.<br>• I do think if it's able to come to the infant's chart that will enhance, a pediatrician's access and, and viewing of it, but I know sometimes that it is not always allowed because it's mom's material. It's mom's pathol, specimen and so it may need to say in maternal chart.<br>• It would be nice if it, you know placenta actually belongs to the baby, and it would be nice if it actually ended up in baby's record.<br>• Um, no, I think oh, actually, so the only other thing I think would be beneficial is like you had alluded to before, having the placental pathology pop up either in baby's chart or whenever we do get a surgical, not a surgical pathology, but when we like have a note input or in a regular lab section if something is new or something is abnormal, they pop up on our screen as a new report being generated which reminds us that there is a report, so if something is taking a week to come back, we sometimes forget to check every day to see if it's back. So, if it could either pop up on the baby chart or have something that show up, then that the pathology report is ready, then that would be helpful.<br>• but how do we link that with you know, mom and baby, because you know, going back and forth, I mean, it might be overlooked, and I was thinking that just in talking to you I may

		have overlooked some results. Having not back gone to mom at another interval.
**Subtheme 1b:** Difficult to understand		• Well, I don’t really understand pathology too much, so I usually look at the executive summary part.<br>• I mean, honestly, I didn’t get a lot of training in residency on how to interpret a placental report, and it’s not something that has come up a lot since I started practicing.<br>• I'm gonna have to say probably most, the majority of the time there's something in there that I don't fully understand. OK, being that I'm not a pathologist.<br>• I would say I read mostly the description or the kind of assessments of it. There have been times I would review other parts if there was something there I didn't quite understand...some of it, I guess, is outside of kind of my knowledge base I guess, and I'm not sure you know how that would apply necessarily to my practice, just not. I guess that would be the biggest part, not part of my knowledge base and that sort of thing...Yeah, in terms of I guess the overall assessment of the of the pathology as opposed to particular details that may or may not, I may or may not understand the significance.<br>• I think early on, definitely there were some words that I did not understand. I would always look it up and they would be pathological terms that it's not coming into my head, but there would be pathological terms that I wouldn't be aware of, so I would just look it up.
**Subtheme 1c:** Doesn’t come fast enough.		• Honestly I don’t do much with the information since it’s after the fact.<br>• By the time I have the report, the mom and baby have been discharged.<br>• Because it’s not available in the time I would need to use the information to take care of my patients, it just never comes to my mind to consider it…it’s not information that is readily available to us in the time that we would need…<br>• So, you know the newborn nursery has such rapid turnover that we have our babies only with us for 24, maximum 72 hours and so if a report comes back four days or five days after delivery, but the baby is fine, we tend to ignore it.<br>• the reports don't come back fast enough for me to ever have seen them while I'm working in the nursery on that particular day.<br>• I think it would be ordered from the obstetrician and we would get a call several days later, saying that perhaps there was chorioamnionitis amnionitis found on the placenta and at that point we’d have to decide what to do for the baby. The

		way infants are leaving the hospital now, they're, they would be at home by the time we got that report.<br>• but especially because there's a fair amount of delay between getting the pathology report and most of the time the patients already discharged from the hospital with the baby. I don't know that there's a lot of, um, that the reports have a lot of impact in my clinical practice.
**<u>Theme 2:</u>** Clinicians value placental pathology for explanatory capability as well as for contributions to current and future care.		
Placental pathology can help explain delivery events and fetal circumstances		• The information can also be useful if we're looking for causes for growth restriction. If the placenta can be a cause of it, we feel more comfortable in assigning that etiology to the growth restriction and don't necessarily have to continue to worry about or track down metabolic or genetic disorders.<br>• if there was an abnormality in the placenta that could um help understand the etiology of something going on with a preemie baby that perhaps was growth restricted.<br>• I generally like to know if the placenta is, is overall normal and appearance so or if there's any gross pathology, especially in a growth restricted baby.<br>• And then the other clinical scenario where I've wanted a placental report is when a baby is very small for gestational age or and and we want to see if the placenta was also really small or placenta was normal size, because a very small placenta could indicate there was issues with mom providing nutrition and providing support for the baby and not necessarily the baby has a chromosomal abnormality or something else.<br>• We expect a pre term placenta to have to not be mature like you would expect that when you see that it is that is a little odd when it has calcifications when it's pre term, but that would explain like growth restriction or if she was hypertensive you can kind of link those things together.<br>• if there is a small baby, concern, for high blood pressure in the mother, um, the placenta does show, you know, the effects of preeclampsia or high blood pressure in the placenta within parts. That's when I do tell the family you know your placenta had the same, that's the reason why the baby had placental insufficiency, so I have done in the past.<br>• and then infants that we don't have a really good reason for growth restriction, that's a big one that I also would review in the past to see you know was the placenta small was there clots on mom's side, fetal side, things of that sort.<br>• Well, I use it to confirm what the clinical scenario was so if I had a patient with ah, well, let's just go with fetal growth restriction. I look and see was the placental, was the placenta

		small, were their reasons. stated in the diagnoses that went along with the clinical scenario.<br>• if I'm told that there are signs of potent…, potentially chorio, then I, we have to make a decision about what to do for the baby in terms of ruling out sepsis.<br>• Usually if it's a baby that I'm concerned about early onset sepsis and I'm trying to gather risk factors that would put that baby at a higher... essentially, the baby may be having symptoms without a clear blood culture so in that scenario, it would be helpful to know if the mom had chorioamnionitis on the placental pathology.<br>• Sure, usually it's in the terms of sepsis. I think when a baby has a really high white count that keeps climbing. So usually we can do a 48 hour rule-out for a baby that's acutely concerning for sepsis, but after 48 hours, maybe the baby's blood cell count is rising or there's minor concerns for sepsis but not so much that we're convinced it's sepsis and so sometimes placental report in those cases help us guide one way or the other - whether this is truly an infection in the baby, or whether this mom had some infection that was maybe having a systemic inflammatory response, but the baby itself might not have an infection, so the clinical cases I'm thinking of are all usually related to sepsis.<br>• Yeah, because a lot of decisions we make are in the beginning, the first few hours of life we have tons of full-term baby that could otherwise look healthy, but because of one very small thing like they're breathing a little fast or they had one temperature being high that they end up getting admitted for rule out sepsis and um, so those times we, if we had a very reassuring placental pathology it might shorten our antibiotics or maybe the baby will be able to go home with mom at 36 hours of life instead of having a longer period of antibiotics and possibly keeping the baby in the hospital one night longer than we normally would have.<br>• if there was a history of infection, sepsis in the baby that I'm trying to look out for sometimes, some babies have morbid courses, which is not very expected at their gestational age or the time. Then we tried to see if there was anything in the placental pathology that we look for. So those are all for infection side. Sometimes there are other, so I think that's the major thing that I've definitely looked out for in placental pathology.<br>• if we've had a demise or a poor uhm, neonatal outcome we'll sometimes look at the placental report to see if there's any like hint to what was going on, especially if it was unclear, you know at the time of the demise or at the time of the delivery, why, you know what was going wrong.

		• sometimes the pathologists would make a comment about the insertion of the cord and how that made a difference and so perhaps if I was seeing early anemia of really unclear etiology, the placental pathology might help us understand a little bit better.<br>• I think more typically it helps us has a better understanding of what caused the perinatal presentation<br>• What I like to do is correlate, read through the placental pathology, look and see if the problem may have originated during prenatal care or very early in the pregnancy. I want to see if there are any indications of some sort of problem, especially prenatally from the placenta that maybe I did not recognize or that was not appreciated in terms of how, how serious it was or how extensive it was.<br>• then the last thing I look for is when there's a a death like a demise, then I'm looking for anything in the report that could indicate why the baby died so then I might read it more detail.
When available, can be useful for antibiotic management		• Um, for me the the most immediate relevant was probably evidence of chorio or infection, because, um, that's something that is, that we can like treat or assess for baby and mom while they're still in in a hospital.<br>• I think again, it's usually when there's funisitis, meaning like an infection on the baby' side that we see on the placenta, and if the baby had any sort of clinical suspicion then I would lean more towards treating the baby with antibiotics longer. So sometimes the diagnosis of funi..., or the description of funisitis pushes me to continue antibiotics longer than I would have otherwise.<br>• Well, if I if a baby has documented chorioamnionitis on the placenta, it may lead me to continue antibiotics for a longer period of time.<br>• I would extend an antibiotic course mostly in those situations if there was evidence of chorioamnionitis.<br>• With regards - infection, it might guide us in how aggressive to be with the infection evaluation and it may impact how long - we frequently start empiric antibiotics in babies because of their clinical presentation - the placenta may in some instances factor into a decision about how long we continue antibiotic therapy.
It's useful for planning future pregnancies		• Well I alert the patient what the findings were and then counsel accordingly if needed.<br>• Well, it doesn't for that pregnancy that's past, but for future, you can counsel you know, accordingly, based on the findings.<br>• Mainly it's to maybe find out things that I need to follow up for the next pregnancy.

	• I sometimes will set up preconceptual counseling for the next pregnancy if they, if there's preeclampsia, an infarction, or you know that it just was a terrible outcome. And If I saw something on the placenta, yeah, it all incorporates for my mind to say you need to go and before you have another planned pregnancy - to plan the pregnancy - and to get in with [MFM] so you know what to expect with another pregnancy like baby aspirin or what have you. There's all sorts of things.<br>• But I use them primarily when trying to answer questions about the pregnancy that just ended to provide some closure, but then also for planning in the future.<br>• It's probably not extremely common that I will look at a placenta and I will say that you know, I'm going to somehow manage your next pregnancy differently other than maybe to just get a general feel for the fact that there were factors that were leading to, um, some sort of placental dysfunction and we try maybe especially hard with the next pregnancy to optimize nutrition, stop smoking, etc.<br>• Um, again, preeclampsia is not something that can be prevented, but definitely having families aware, making sure that they seek attention. I think that is still good and also for them to know like it's not their fault. It's not that mom's nutrition was not good or something like that, so just to have, I think it benefits family and it definitely helps us counseling them.<br>• And then long term, some very specific placental lesions may have a risk for recurrence and so I look for those as well, in a future pregnancy...<br>• I think it influences it in the sense that if I'm doing preconception counseling on a woman who had like an IUFD or something like that I will use all the information including placental pathology, to kind of help her with the next pregnancy. I don't think it changes much, but it kind of it, I think it adds to the counseling.
**Aim 2: Explore with maternal and neonatal care providers what information could improve clinical care for mothers and newborns that might be ascertained from placental data.**	
**Theme 3:** Providers would like basic information quickly from the placenta: weight, completeness, presence of signs of infection, infarcts.	• when they're talking about the placental size if the grammage could just go up in the top, in the impression where it says like you know average for gestational age, I like to just see what the number is, so if the number could be included up there that would be really cool.<br>• I think the weight of the placenta matters to me. Usually I think of placenta as being a third of the babies weight, so sometimes I use that to gauge whether the placenta was big or small.

		• [I'm looking for] size of the placenta.<br>• Oh, in the first hour placental weight for gestational age, are there any, um, that's a tough question in the first hour, yeah, maybe placental weight for gestational age.<br>• [I would like to see] A weight, and if there was an infection, and infarction.<br>• [I would like to see] I think like infection and infarction/abruption you know, those two things.<br>• [I would like to see] Anything that looked retained or any signs of infection.<br>• I guess if the whole thing's out or not, if it looks like it's intact. I mean we, we look at it grossly in the delivery room, obviously missing but, I mean if there was something clearly missing when…<br>• The appearance tells me whether or not we need to keep an eye on the patient for postpartum hemorrhage. So if I suspect that there's a little bit of a placental piece that's missing, then I know that we need to watch for hemorrhage, or if it looks like there's a piece that may have been missing, we can watch for infection.
Rapid placental pathology could inform risk stratification and clinical decision making for antibiotics		• I guess an indication or a scale, maybe not a scale, but some sort of indication that a finding is extremely rare or pathologic 'cause sometimes finding, I don't know like high white cell or certain things might sound bad, but it's like found in 80% of placentas and it probably doesn't mean as much so it would be helpful if something is suggestive of pathology or something along those lines.<br>• I would like a general idea if that placenta looks like it was essentially normal, if that placenta overall appears to be generally what's seen with a normal delivery with a normal infant, or if there is anything that, and I wouldn't need detail, but you know, to have something that says this doesn't look quite right.<br>• I think just whether the overall health I guess I don't see I don't really wouldn't know how to describe that…but the overall health of the placenta and maybe…clinical definitions and things of that sort would be helpful with that, because I think that would guide the work up moving forward.<br>• I wouldn't necessarily go crazy about involving genetics or you know other things that may or may not be relevant, if it was, you know pretty obvious that the placenta itself was pretty sick, though it would, it could affect my management and in terms of further work up.<br>• If there is a way that information about infection can help us stratify the newborn's risk, that would probably be the most helpful, because that's what we're usually trying to figure out in the 1st 24 hours. Does the baby need

		antibiotics? Can we just monitor them? I don't know if that like is actually available though if that if there's evidence behind this {inaudible} information to determine that.<br>• Certainly infection, if they have concern for infection in the placenta, then we should know so that we can figure out what we want to do from an infant standpoint.<br>• If it would come back relatively quickly to confirm or rule out infection, that would make a difference in terms of management, particularly for the baby. In terms of needing antibiotics or not.<br>• I would use it more. And it will help me to make a decision about continuing antibiotics, particularly if the blood culture is negative and the baby looks or act like septic baby. Then I would, if the pathology supports the infection, inflammation, then I would treat the infection as such. So, I expect, I would expect to use more.<br>• So, I would say infection any obvious signs of infection, chorio, um, that would be really helpful for the baby.<br>• Yeah, because a lot of decisions we make are in the beginning, the first few hours of life we have tons of full-term baby that could otherwise look healthy, but because of one very small thing like they're breathing a little fast or they had one temperature being high that they end up getting admitted for rule out sepsis and um, so those times we, if we had a very reassuring placental pathology it might shorten our antibiotics or maybe the baby will be able to go home with mom at 36 hours of life instead of having a longer period of antibiotics and possibly keeping the baby in the hospital one night longer than we normally would have.<br>• if there was an infection. If there was a clear like bacteria that was associated like could we have changed our antibiotic management.<br>• But if the, the baby. We're having any issues that might be important for early diagnosis. The same thing for mom that that if the mom develops a fever postpartum and their placenta's floridly infected or or you know you knew that had a lot of inflammation. Then you might be quicker on your trigger to start antibiotics or something like that so.<br>• And it will help me to make a decision about continuing antibiotics, particularly if the blood culture is negative and the baby looks or act like septic baby. Then I would, if the pathology supports the infection, inflammation, then I would treat the infection as such. So, I expect, I would expect to use more.<br>• A scenario where it would be helpful is that if a bacteria came back positive from the placenta or there was diagnosed chorio and the baby potentially had a negative culture but continued to be sick, and perhaps I may continue antibiotics

		longer than 48 hours, 72 hours, maybe even prolonged depending on that and that information. Again, it doesn't happen that frequently, and again, because our culture on the baby is the gold standard.<br>• I think it would help guide us in terms of the antibiotic treatment for our infants. We try to minimize antibiotic exposure now, as best that we can, which is a big practice change from, you know, when I started, so that has actually been quite helpful in that situation...
**Theme 4:** Placental pathology reports that connect clinically relevant findings and that are written with plain, standardized language are preferred.		• Well, I I didn't know, let me think about it. I guess it could be so when I think of your pathology report. For me the most, the closest thing that I'm thinking of is actually a Pap smear report. And Pap smear reports have it's very, so there, they don't tell you how to manage the patient. However, they do say negative. They do say high risk. They do certain terminologies that you would probably need, so I would I would have. I would really appreciate that. 'Cause right now it is truly very generic, it is just it is information, but sometimes people who don't work, you know, they just don't see the pathology as much as some other people do and I think that it can, some things can be missed.<br>• To be clinician friendly I think for the average person. It's going to have to have some kind of interpretation of the lesion. So, if they say something like, um increase syncytial knots that may not mean something, it means something for me, but for the average person they could say something like this is a sign of placental underperfusion, you know and said that would help the clinician say, oh, okay, so this mom didn't have a good implantation.<br>• I think for me it would be really helpful to to get to get more information as opposed to just the objective findings, like the possible implications this could have on outcomes and management recommendations.<br>• So I always love when our specialists in, or radiologist or the pathologist, include a line at the bottom that says, you know, these are the findings we see on the on the slide or on the studies, and it could indicate this clinical scenario and then they detail consider this, this, this and this as your clinical entity, I really find that helpful, especially for things that aren't occurring very often or variants that I've never seen before. I really appreciate that point in a in a direction.<br>• Well, I think from a standpoint of the user, having a clear and fairly concise summary of the findings and what is clinical relevance maybe would be very, very helpful. I do think there is value to having the technical aspects of the placental report, but if we're looking at just like a, immediately available tool to use that would certainly, that would almost be like a footnote, but the main highlighted text would be

		what are the, what’s the major finding and what is its potential clinical relevance.<br>• I might ask for some pathologic correlations, that if certain, histologic findings, if those were associated with certain prenatal conditions or infant conditions, that’d be great, you know, sort of a little minimized pathology textbook.<br>• So almost a differential diagnosis of when you do see these types of lesions…yes, that would be very helpful.<br>• I think definitely trying to have some clinical significance or association. Sometimes the pathology report is like this is what was found, but I've seen some pathologists actually say, like you know this is more common with this disease, so there is some clinical significance which is very helpful because as a pathologist I think they are the experts who have seen more like placenta or, so, I think that is, is very helpful to have included in the report.<br>• Is it just use like words that are more, easier to understand, I mean the sometimes like I just this and I just that, like is there evidence of infection, is there evidence that it's intact?<br>• I've noticed that different pathologists will use different terminology for basically the same finding, and so standardization of the terms that they're using would be fine. Part of me would like it to be more plain language so a non-pathologist would be more readily able to read it. But at the same time, I I kind of feel a little guilty about that, like I'm a doctor, I should probably know what these words mean. You know we're not, we’re not writing a children's book, um, but that I think I think at least standardization would be a little bit helpful, so that I'm not encountering a new way to describe the same problem. That would probably be the biggest thing.<br>• That would be, yeah, if the definition of the term was right there on the report that I wouldn't have to take the extra time to look it up.<br>• I think it would be helpful to know what the definitions are, so if it's written in a report, I know how to how to take that information. So perhaps if someone writes something and I think it has a different definition, I think I want to know what definition is being used.<br>• I don't know, um, I guess the biggest thing I would want included obviously were the main diagnoses, but I would want it worded in a way that I didn't have to go hunting for what the terminology actually means. So, my most important thing would be something that's in plain English.<br>• Well, I, I think it would be helpful, but I think it's probably more helpful from the pathologists of standpoint that they don't always agree on diagnosis and standardization. So, so

		that's really a big problem with placental pathology so, uh you know they would have to say some, let's just take, uh, increase syncytial knots, so they would have to say something like this is diagnosed when certain percentage of the villi in certain gestation age show these findings so I think that standardization might be helpful for pathologist because I don't think that they're standardized and that's a limitation of placental diagnosis and they don't all have the same either interest or ability in placental pathology. So there's a wide range of different pathologists may pick up on something and some may not…
Some expressed concern about connecting outcomes to a diagnosis		• I don't think that's as helpful, because I think that the pathology report is a data piece and it might, ah, I mean, I, I think it should be used by the appropriate providers, but I don't, if you suggest potential outcomes, I think you could potentially go down other pathways that are maybe not as beneficial.<br>• I'm always leery about making things real cookie cutter because I feel like it, it gives a false sense of security. I think using the syncytial knots as an explanation, like explaining what that means and then saying like this is oftentimes seen in cases of hypertensive disorders of pregnancy and then leaving it at that. Yeah, 'cause I'm kind of concerned that if you start saying like. Ah, your patient may have blah blah blah blah blah like that's it goes down a really slippery slope.

[ ] indicates paraphrase
