# Supplementary material for: Placental pathology reports: A qualitative study in a US university hospital setting on perceived clinical utility and areas for improvement
Source: PLoS One. 2023 Jun 8;18(6):e0286294. doi: 10.1371/journal.pone.0286294 (PMC10249791; doi:10.1371/journal.pone.0286294)
Supplement: S2 Data — (PDF) [file pone.0286294.s002.pdf]

Name, Code Type, Folder Location, List Level, List Order, Aggregate, Nickname, User Assigned Color
1. Accessibllity and timing, Code, Codes, 1, 1, False, , None
1a. How providers get results, Code, Codes, 2, 2, False, , None
1b. How patients get results, Code, Codes, 2, 3, False, , None
1c. Report utilization, Code, Codes, 2, 4, False, , None
1d. Training in interpretation, Code, Codes, 2, 5, False, , None
1e. Timing of report, Code, Codes, 2, 6, False, , None
1f. Other, Code, Codes, 2, 7, False, , None
2. Current report utilization - what are we looking for to care for, Code, Codes, 1, 8, False, , None
2a. Mom, Code, Codes, 2, 9, False, , None
2ai. counsel for future pregnancy_future maternal health, Code, Codes, 3, 10, False, , None
2aii. care or intervention for mother, Code, Codes, 3, 11, False, , None
2aiii. medical problems, Code, Codes, 3, 12, False, , None
2aiv. to explain or confirm clinical presentation_situation, Code, Codes, 3, 13, False, , None
2av. Placenta normal or not, Code, Codes, 3, 14, False, , None
2avi. Other, Code, Codes, 3, 15, False, , None
2b. Baby, Code, Codes, 2, 16, False, , None
2bi. confirm clinical presentation, Code, Codes, 3, 17, False, , None
2bii. growth of baby, Code, Codes, 3, 18, False, , None
2biii. placental weight, Code, Codes, 3, 19, False, , None
2biv. twinning - type of placentation, Code, Codes, 3, 20, False, , None
2bv. impacts antibiotic management, Code, Codes, 3, 21, False, , None
2bvi. determine future testing, Code, Codes, 3, 22, False, , None
2bvii. understanding poor outcomes, Code, Codes, 3, 23, False, , None
2bviii. other, Code, Codes, 3, 24, False, , None
2c. Common clinical conditions for requesting placental pathology, Code, Codes, 2, 25, False, , None
3. Future report utilization, Code, Codes, 1, 26, False, , None
3a. Most important information to have quickly, Code, Codes, 2, 27, False, , None
3ai. placental weight for gestational age, Code, Codes, 3, 28, False, , None
3aii. general findings, Code, Codes, 3, 29, False, , None
3aiia. normal, Code, Codes, 4, 30, False, , None
3aiib. not normal, Code, Codes, 4, 31, False, , None
3aiii. infection, Code, Codes, 3, 32, False, , None
3aiv. twinning information, Code, Codes, 3, 33, False, , None
3av. placentation, Code, Codes, 3, 34, False, , None
3b. Wishes for future report, Code, Codes, 2, 40, False, , None
3bi. interpretation of lesion, Code, Codes, 3, 41, False, , None
3bii. diagnosis with outcomes correlation, Code, Codes, 3, 42, False, , None
3biia. link to outcomes, Code, Codes, 4, 43, False, , None
3biib. do not link to outcomes, Code, Codes, 4, 44, False, , None
3biii. liklihood of recurrence, Code, Codes, 3, 45, False, , None
3biv. definitions_standardization of terms, Code, Codes, 3, 46, False, , None
3biX. timing_continuity of care, Code, Codes, 3, 53, False, , None
3bv. management recommendations_postpartum managemtn, Code, Codes, 3, 47, False, , None

3bva. link to recommendations, Code, Codes, 4, 48, False, , None
3bvb. do not link to recommendations, Code, Codes, 4, 49, False, , None
3bvi. findings_description, Code, Codes, 3, 50, False, , None
3bvii. link to literature or citation, Code, Codes, 3, 51, False, , None
3bviii. report characteristics, Code, Codes, 3, 52, False, , None
3bX. overall risk assessment, Code, Codes, 3, 54, False, , None
3ix. intact, Code, Codes, 3, 38, False, , None
3vi. congenital_genetic abnormality noted, Code, Codes, 3, 35, False, , None
3vii. infarction, Code, Codes, 3, 36, False, , None
3viii. abruption, Code, Codes, 3, 37, False, , None
3x.other, Code, Codes, 3, 39, False, , None
